# Chemical diversity of microbial volatiles and their potential for plant growth and productivity

**DOI:** 10.3389/fpls.2015.00151

**Published:** 2015-03-13

**Authors:** Chidananda Nagamangala Kanchiswamy, Mickael Malnoy, Massimo E. Maffei

**Affiliations:** ^1^Research and Innovation Center, Biology and Genomic of Fruit Plants, Fondazione Edmund MachTrento, Italy,; ^2^Plant Physiology Unit, Department of Life Sciences and Systems Biology, University of TurinTurin, Italy

**Keywords:** microbial volatile compounds, bacteria, fungi, plant productivity, sustainable agriculture

## Abstract

Microbial volatile organic compounds (MVOCs) are produced by a wide array of microorganisms ranging from bacteria to fungi. A growing body of evidence indicates that MVOCs are ecofriendly and can be exploited as a cost-effective sustainable strategy for use in agricultural practice as agents that enhance plant growth, productivity, and disease resistance. As naturally occurring chemicals, MVOCs have potential as possible alternatives to harmful pesticides, fungicides, and bactericides as well as genetic modification. Recent studies performed under open field conditions demonstrate that efficiently adopting MVOCs may contribute to sustainable crop protection and production. We review here the chemical diversity of MVOCs by describing microbial–plants and microbial–microbial interactions. Furthermore, we discuss MVOCs role in inducing phenotypic plant responses and their potential physiological effects on crops. Finally, we analyze potential and actual limitations for MVOC use and deployment in field conditions as a sustainable strategy for improving productivity and reducing pesticide use.

## Introduction

Volatile Organic Compounds (VOCs) typically occur as a complex mixture of low-molecular weight lipophilic compounds derived from different biosynthetic pathways. To describe their complexity the term “volatilome” has been recently proposed (Maffei et al., 2011). In nature, VOCs are responsible for inter- and intra-organismic communication, partaking in innumerable interactions between plants, antagonists, and mutualistic symbionts both below and above ground (Maffei, 2010; Maffei et al., 2011; Garbeva et al., 2014b; Lemfack et al., 2014; Kanchiswamy et al., 2015).

Volatile Organic Compounds can travel far from the point of production through the atmosphere, porous soils, and liquid, making them ideal info-chemicals for mediating both short- and long-distance intercellular and organismal interactions (Maffei et al., 2011). In the past there was less focus on volatiles of microorganisms with respect to VOCs from species of the plant and animal kingdom(Stahl and Parkin, 1996; Schulz and Dickschat, 2007; Korpi et al., 2009; Effmert et al., 2012; Junker and Tholl, 2013; Weisskopf, 2013; Penuelas et al., 2014).

The cosmopolitan distribution of microorganisms creates a context for frequent and frequently overlooked, biotic responses to microbial emissions. In some ecosystems, bacterial or fungal emissions can also incite biotic aggregations, and often a single microorganism or emission can have different effects on biota behaviors, especially across species, ontogenies, and habitats (Davis et al., 2013). These interactions prompt coevolution, whose process among biota – including viruses, fungi, bacteria, plants, nematodes, insects, and mammals, is considered by many biologists to have generated much of the earth’s biological diversity (Occhipinti, 2013).

Microbial volatile organic compounds (MVOCs) are a type of VOCs produced by all microorganisms as part of their normal metabolism. They serve as chemical windows through which the fundamental information about the molecular basis of microbial activities is released (Liang et al., 2008; Korpi et al., 2009; Thorn and Greenman, 2012). There appears to be a multipartite basis for organisms responses to MVOCs, and complex trophic interactions can result from the production of MVOCs. Moreover, species-specific MVOCs may also serve as marker compounds for the selective detection of fungal and bacterial species in the environment (Fiedler et al., 2001). Other transformations may, however, occur in detoxification processes (Marmulla and Harder, 2014). Many of the formed MVOCs are produced by soil microorganisms, and it would be a challenge to investigate soil microbial communities by studying their MVOC profile (Insam and Seewald, 2010). To date, 100s of bacteria and fungi were described as soil MVOC producers (Effmert et al., 2012).

The comparative analysis of experimental data has shown that volatile metabolites make a much greater contribution to the microbial interactions than non-volatile ones. It has been found that interaction of microorganisms via the volatiles they release occurs frequently and is typical of a number of microorganisms (Tirranen and Gitelson, 2006). Understanding of the MVOCs, as well as the insights into the molecular basis associated with the MVOCs, can provide a real-world capabilities for better control and utilization of microorganisms (Liang et al., 2008). Systematic exploration of MVOCs and the characterization of their biological functions and ecological roles will likely uncover novel mechanisms for controlling diverse biological processes critical to plant health and will also offer tangible practical benefits in addressing agricultural and environmental problems (Bitas et al., 2013).

**FIGURE 1:**
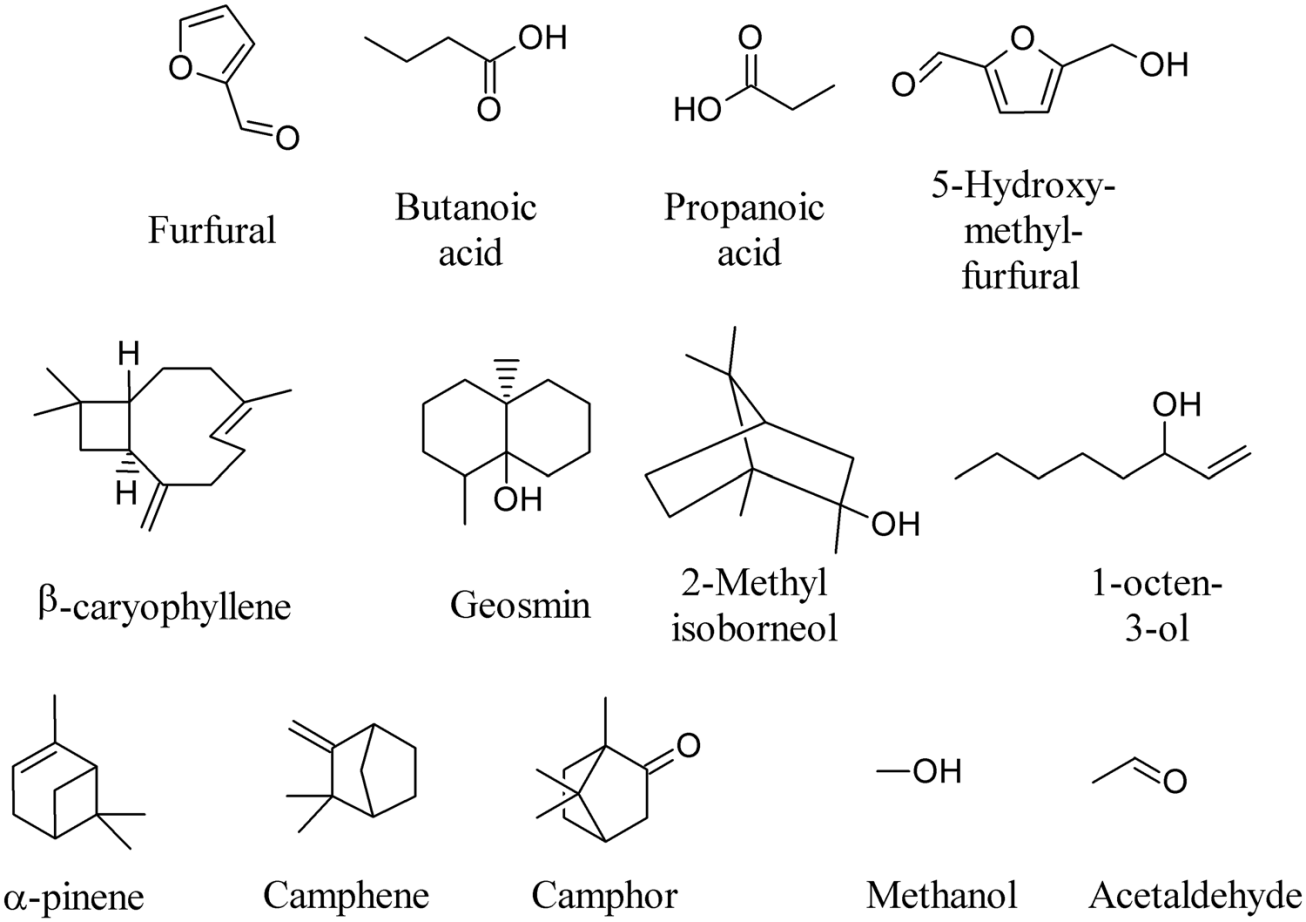
**Most frequently emitted Microbial volatile organic compounds (MVOCs)**.

Volatile metabolites released by microorganisms produce an inhibitory, sometimes bactericidal, effect on the vital functions of bacteria, while the stimulating action occurs 6–8 times less frequently (Tirranen and Gitelson, 2006). To quote a few examples, furfural, butanoic acid, propanoic acid, 5-hydroxy-methyl-furfural, β-caryophyllene, geosmin, 2-methyl isoborneol, 1-octen-3-ol, α-pinene, camphene, camphor, methanol, and acetaldehyde (**Figure 1**) are among the most frequently emitted compounds (Stahl and Parkin, 1996; Li et al., 2004; Muller et al., 2004a,b; Leff and Fierer, 2008; Gray et al., 2010; Insam and Seewald, 2010; Ramirez et al., 2010; Wenke et al., 2010; Perl et al., 2011; Juenger et al., 2012; Sundberg et al., 2013).

Recent developments in analytical instrumentation and bioinformatics software have established metabolomics as an important research tool for studying ecological interactions between organisms (Kuske et al., 2005; Scotter et al., 2005; Bunge et al., 2008; Liang et al., 2008; Wihlborg et al., 2008; Mcneal and Herbert, 2009; O’Hara and Mayhew, 2009; LeBouf et al., 2010; Ramirez et al., 2010; Rasanen et al., 2010; Dolch et al., 2012; Juenger et al., 2012; Thorn and Greenman, 2012; Aponte et al., 2013; Kunze et al., 2013), while recent reviews described MVOCs biosynthesis (Davis et al., 2012; Penuelas et al., 2014).

In this review, we will give an overview of the chemical diversity of microbial volatiles and we will address the important issue of the exploitation of these bioactive molecules to improve plant growth, development, and health in a sustainable agricultural context.

## Overview of Bioactive Bacterial Volatiles

Bacteria emit a wealth of volatiles. During the past few years, an increasing awareness concerning the emission of an unexpected high number of bacterial volatiles has been registered and recent investigations have clearly demonstrated that bacteria employ their volatiles during interactions with other organisms in order to influence populations and communities (Kai et al., 2009; Romoli et al., 2014). A wide array of compounds has been identified in bacterial emission of a large number of chemicals. Bacteria are known to either positively or negatively affect other organisms’ fitness and recent studies have suggested that bacterial volatiles play an important role in bacterial–plant, bacterial–bacterial, and bacterial–fungal interactions.

### MVOC in Bacterial–Plant Interactions

Hundreds of different bacterial MVOCs have been identified, comprising alkanes, alkenes, alcohols, esters, ketones, sulfur compounds, and terpenoids. The appearance of a characteristic volatile profile or compound is attributable to the specific metabolism or metabolic pathway(s) active in the bacteria (Kai et al., 2009). Such volatiles are ideal infochemicals because they occur in the biosphere over a range of concentrations and can act over long distances (Wheatley, 2002).

Some bacteria preferentially live in the soil closely associated with the plant roots, exploiting the rich nutrient exudates that plants deliver into the soil. These bacteria are collectively defined rhizobacteria and many of them promote plant growth (Bhattacharyya and Jha, 2012), whereas the root environment they colonize is called the rhizosphere (Mendes et al., 2013). Among rhizobacteria, Pseudomonads have been considered to be important rhizosphere organisms (Goswami et al., 2013). VOCs produced by rhizobacteria are involved in their interaction with plant-pathogenic microorganisms and host plants and show antimicrobial and plant-growth modulating activities (Vespermann et al., 2007). Fluorescent *Pseudomonas* strains help in maintenance of soil health and protect the crops from pathogens (Hol et al., 2013). Rhizospheric bacterial strains can modulate both plant growth promotion and root-system architecture by differential VOC emission (Gutierrez-Luna et al., 2010). Recent analytical developments have provided a most comprehensive profile of rhizobacterial volatiles. These MVOCs exhibit molecular masses below 300 Da and are rather lipophilic with relatively low boiling points.

**FIGURE 2:**
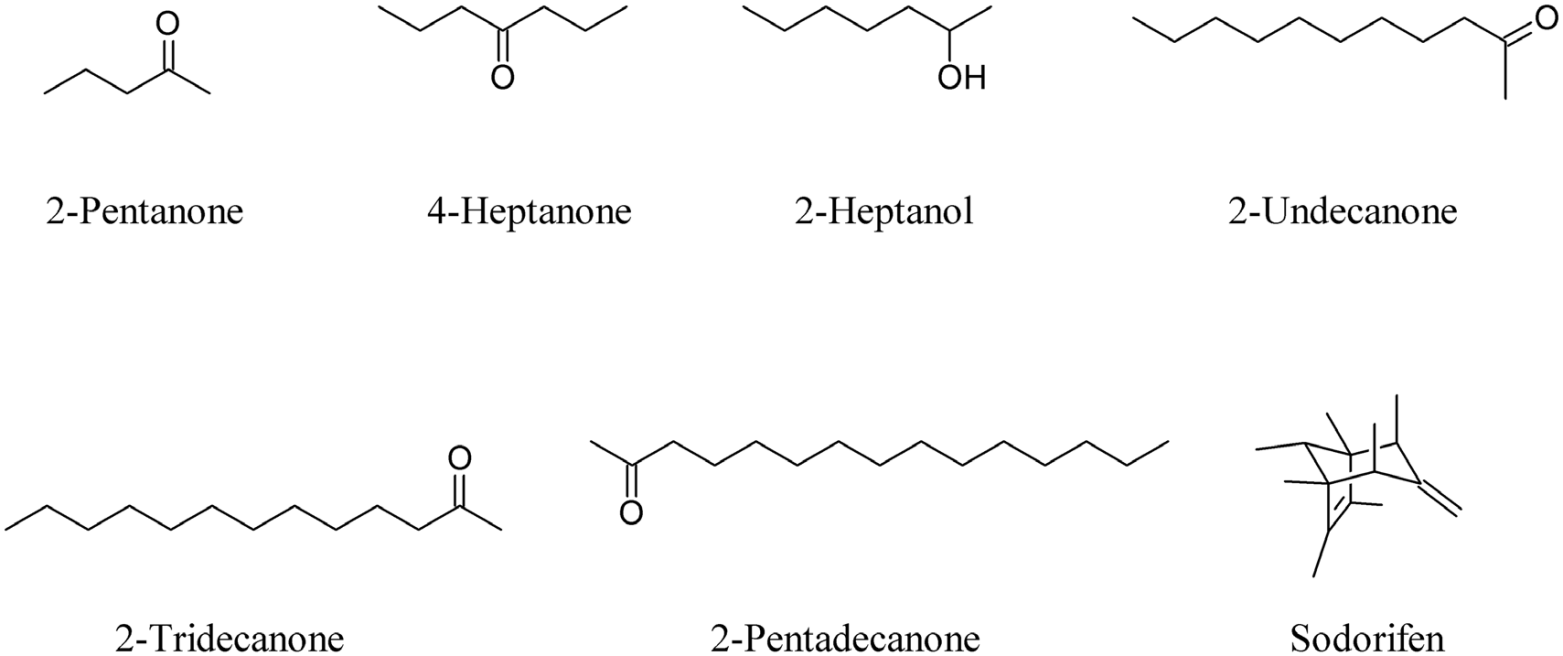
**Common bacterial volatile organic compounds (VOCs) and the bicyclic oligomethyl octadiene sodorifen**.

As well as commonly known bacterial VOCs such as 2-pentanone, 4-heptanone, 2-heptanol, 2-undecanone, 2-tridecanone, and 2-pentadecanone (Schulz and Dickschat, 2007; Weise et al., 2014), well known compounds like sodorifen, a bicyclic oligomethyl octadiene produced by *Serratia odorifera* (Kai et al., 2010), are able to interfere with plants (**Figure 2**).

Forty-two soil-borne bacterial strains were screened for their volatile-mediated effect on 6-day-old seedlings of *Arabidopsis thaliana*. Thirty-six compounds of bacterial origin were selected for further analysis and among these 1-hexanol, indole, and pentadecane stimulated plant growth (Blom et al., 2011). Co-cultivation of *A. thaliana* with *S. odorifera* in bi-partite Petri dishes, which only allowed volatiles to diffuse from one side to the other, resulted in dramatic growth inhibition of plants (Vespermann et al., 2007). Dimethyl disulfide (DMDS) and ammonia are among the most bioactive compounds (Kai et al., 2010).

Application of DMDS produced by a *Bacillus cereus* strain significantly protected tobacco (*Nicotiana tabacum*) and corn (*Zea mays*) plants against *Botrytis cinerea* and *Cochliobolus heterostrophus*, respectively (Huang et al., 2012). Furthermore, DMDS supplementation significantly reduced the expression of *Nicotiana attenuata* sulfur-assimilation genes, as well as methionine biosynthesis and recycling (Meldau et al., 2013). Two compounds, 3-hydroxy-2-butanone also known as acetoin and 2,3-butanediol (2,3-BD), were released consistently from strains of *Bacillus subtilis* and *B. amyloliquefaciens* and were found to significantly enhance total leaf surface area and induced systemic resistance (ISR) of *A. thaliana* (Ryu et al., 2003; Rudrappa et al., 2010). 2,3-BD was also one of the major MVOCs produced by *Enterobacter aerogenes*, an endophytic bacterium that colonizes corn plants. The production of 2,3-BD by *E. aerogenes* rendered corn plants more resistant against the Northern corn leaf blight fungus *Setosphaeria turcica* (D’Alessandro et al., 2014).

**FIGURE 3:**
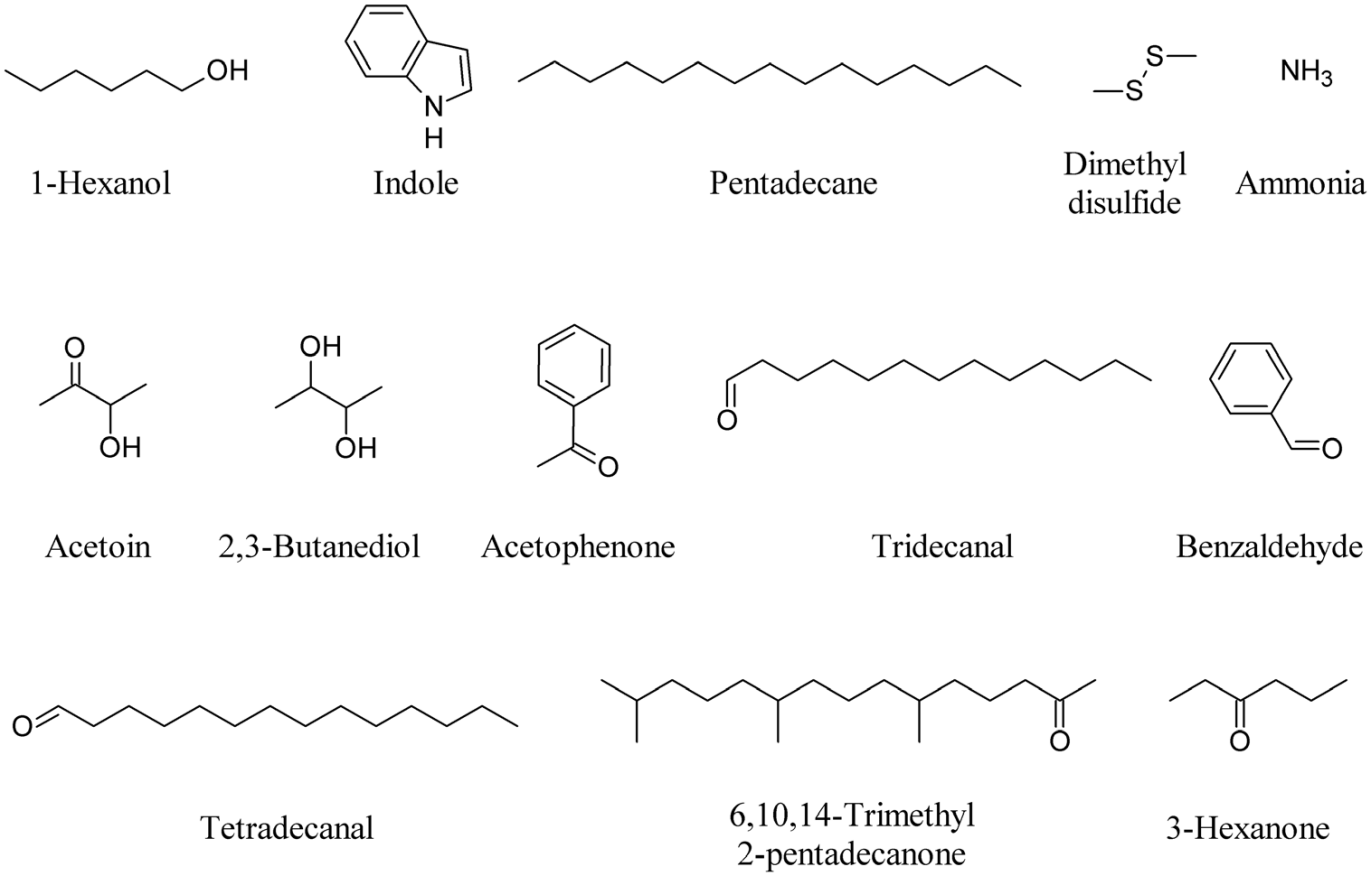
**Bacterial VOCs able to induce plant responses**.

The differential emission of acetophenone, tridecanal, tetradecanal, 6,10,14-trimethyl 2-pentadecanone and benzaldehyde produced by different lemon rhizobacteria showed that the effect observed in *Arabidopsis* roots is proportional to the type and amount of compounds produced by the bacteria (Gutierrez-Luna et al., 2010). 3-Hexanone produced by strains of *Burkholderia ambifaria* significantly increased *Arabidopsis* biomass, as did acetophenone and DMDS (Groenhagen et al., 2013). **Figure 3** depicts some bacterial VOCs able to induce plant responses.

**FIGURE 4:**
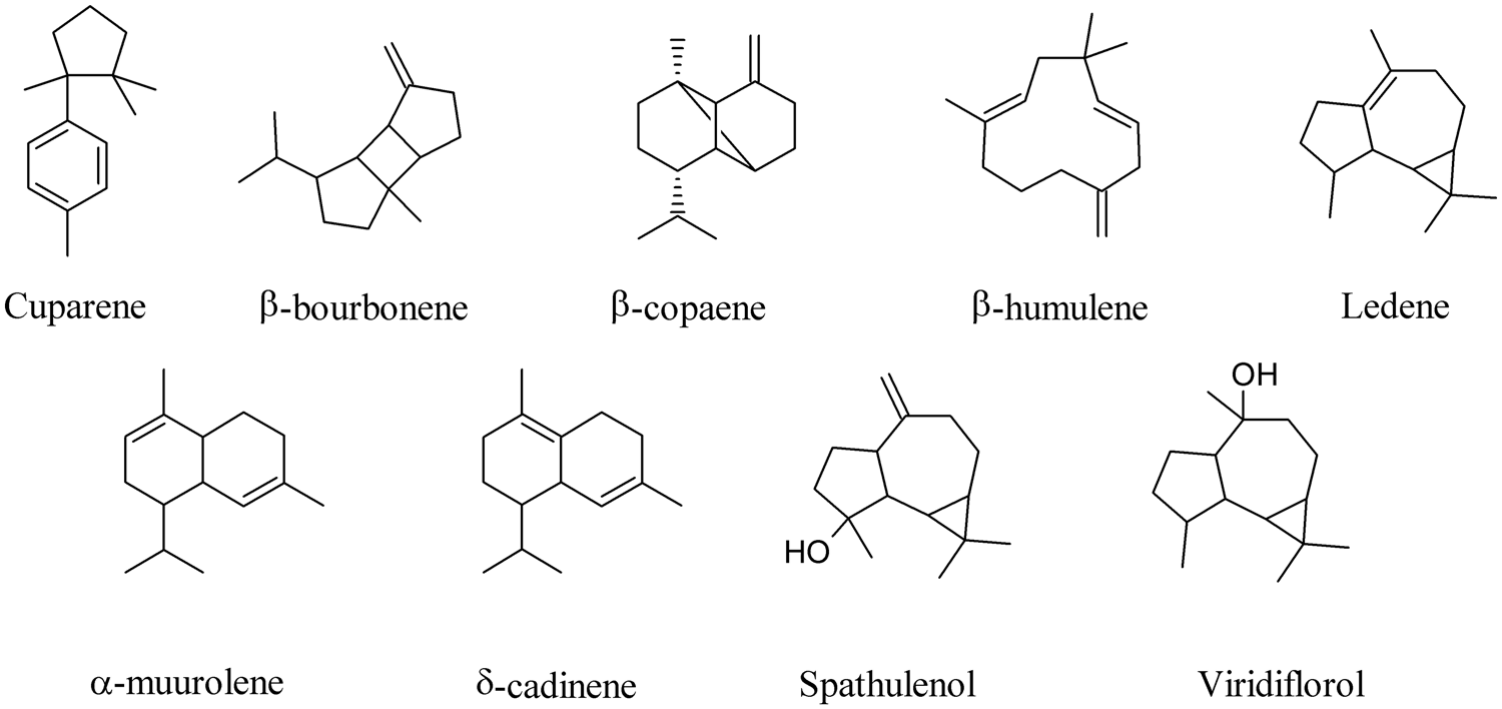
**Biotransformation products of cuparene by Vetiver endobacteria**.

A broad phylogenetic spectrum of bacteria, including α-, β-, and γ-Proteobacteria, high-G+C-content Gram-positive bacteria, and microbes belonging to the Fibrobacteres/Acidobacteria group live inside special cells surrounding the root vascular cylinder of vetiver (*Vetiveria zizanioides*; Maffei, 2002). Most of them are able to grow by using oil sesquiterpenes as a carbon source and to metabolize them releasing into the environment a large number of compounds typically found in commercial Vetiver oils (Del Giudice et al., 2008; Alifano et al., 2010; Maffei et al., 2011). When the sesquiterpene cuparene, for instance, was fed to Vetiver root-associated bacteria an amazing number of other unrelated sesquiterpenes were produced, including β-bourbonene, β-copaene, β-humulene, ledene, α-muurolene, δ-cadinene, spathulenol, viridiflorol (**Figure 4**), and β-caryophyllene (**Figure 1**; Del Giudice et al., 2008). These results underline the ability of bacteria to biotransform *in vivo* complex MVOCs.

### MVOC in Bacterial–Bacterial Interactions

Volatiles of bacteria can influence the metabolism of other bacteria but the role of volatiles in interactions between bacterial species has been studied very little. Given the physically separated distribution of bacterial populations (micro-colonies) in the porous soil matrix it has recently been suggested that MVOCs play key roles in interspecific bacterial interactions (Garbeva et al., 2014a). It is expected that rhizosphere-inhabiting bacteria might invest a substantial part of the energy obtained from metabolizing root-exudates to produce bioactive MVOCs.

**FIGURE 5:**
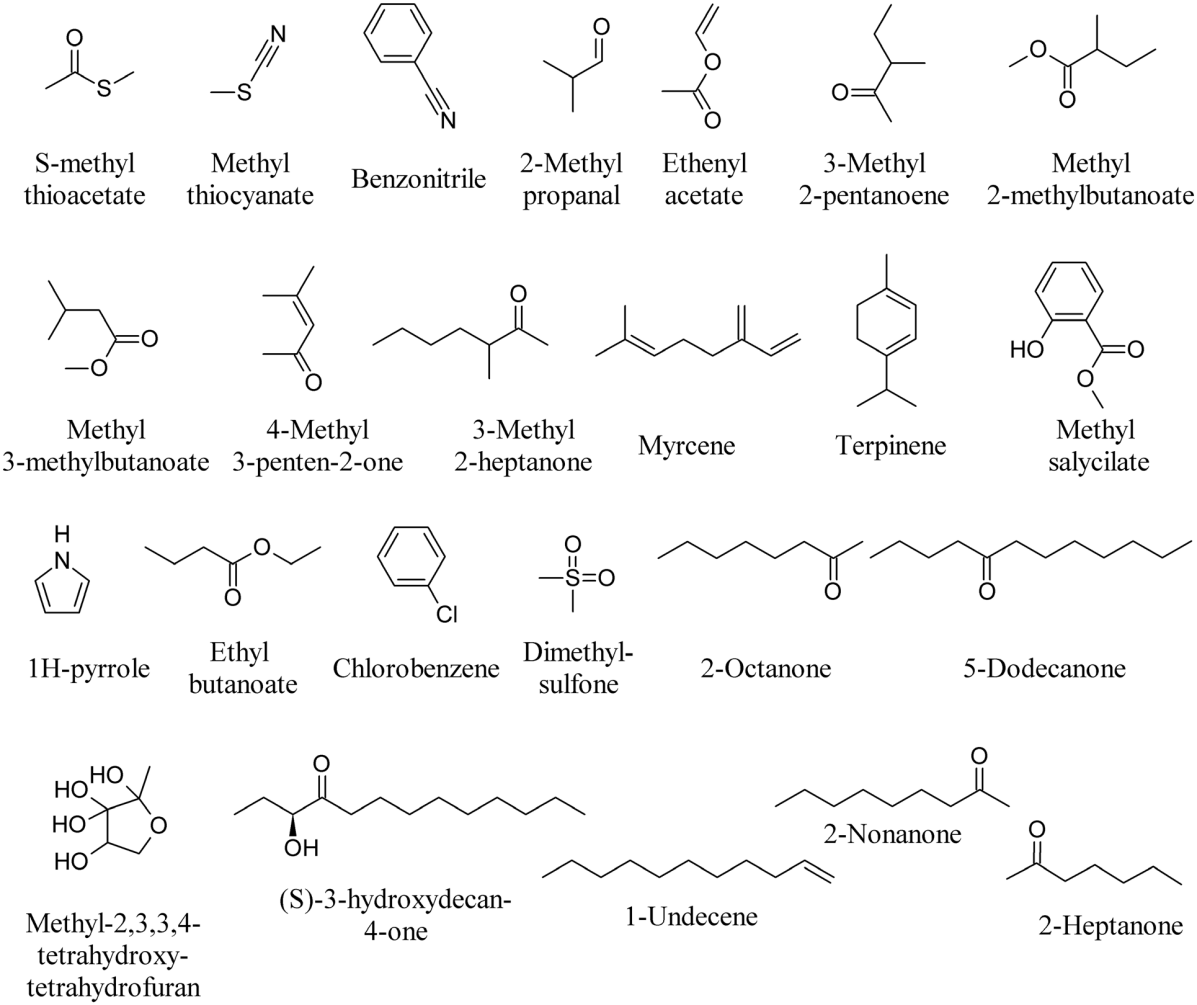
**Bacterial volatiles able to induce bacterial responses**.

Volatiles produced by *Collimonas pratensis* and *Serratia plymuthica* stimulated the growth of *Pseudomonas fluorescens*, whereas volatiles emitted by *Paenibacillus* sp., *Pedobacter* sp. and the mix of all four bacteria did not affect *P. fluorescens* growth (Garbeva et al., 2014a). The highest numbers of unique volatile compounds were emitted by *C. pratensis* and *S. plymuthica*, including *S*-methyl thioacetate, methyl thiocyanate, benzonitrile (**Figure 5**) and DMDS (**Figure 3**). Specific MVOCs produced by *C. pratensis* included among others: 3-hexanone (**Figure 3**), 2-methyl propanal, ethenyl acetate, 3-methyl 2-pentanoene, methyl 2-methylbutanoate, methyl 3-methylbutanoate, 4-methyl 3-penten-2-one, 3-methyl 2-heptanone, myrcene, terpinene, and methyl salicylate (**Figure 5**). Specific MVOCs produced by *S. plymuthica* included among others: 2-pentadecanone (**Figure 2**), 1H-pyrrole, ethyl butanoate, chlorobenzene, dimethylsulfone, 2-octanone, and 5-dodecanone (**Figure 5**; Garbeva et al., 2014a).

It has recently been demonstrated that bacteria are able to inhibit the growth of *Burkholderia cepacia* complex (Bcc) strains through the synthesis of MVOCs (Papaleo et al., 2012, 2013; Orlandini et al., 2014). Reported data show that two *Pseudoalteromonas* strains were able to completely inhibit the growth of most Bcc strains (Romoli et al., 2011). Methyl-2,3,3,4-tetrahydroxytetrahydrofuran (**Figure 5**), indole (**Figure 3**) and its derivatives, quinolones and (*S*)-3-hydroxytridecan-4-one (**Figure 5**) as signals have also been described (Diggle et al., 2006; Ryan and Dow, 2008).

Expressions of phenotypic characteristics in Gram-negative bacteria such as bioluminescence, biofilm formation and production of virulence factors, exoenzymes, antibiotics and pigments are often regulated by a cell density-dependent cell-to-cell communication quorum-sensing (QS) network mediated by *N*-acyl homoserine lactone (AHL) signal molecules (Bassler, 2002; Waters and Bassler, 2005; Ng and Bassler, 2009). AHLs are used by Gram-negative bacteria to monitor population density, a term commonly referred to as QS (Ortiz-Castro et al., 2008). AHLs belong to a class of bacteria-produced amino compound-containing lipids. AHL signals have been described in many plant-associated bacteria, including plant growth-promoting rhizobacteria (PGPR; see below). The ability to disrupt QS networks is termed quorum quenching (QQ), and is an important mechanism of competition between bacteria (Chernin et al., 2011). An elucidation of the mechanisms governing the QQ phenomenon might help in developing new approaches to controlling plant pathogens (Rasmussen and Givskov, 2006). AHL-mediated communication between individual bacterial cells has been detected in the rhizosphere, and rhizospheric bacteria have been shown able to persist and produce MVOCs inside the plant.

Microbial volatile organic compounds produced by some *P. fluorescens* and *S. plymuthica* strains inhibited the growth of *Agrobacterium tumefaciens* and *A. vitis* strains *in vitro*. DMDS was the principal headspace volatile produced by *S. plymuthica*; it strongly suppressed *Agrobacterium* growth *in vitro* and was emitted by tomato plants treated with *S. plymuthica*. 1-Undecene (**Figure 5**) was the main volatile emitted by the *P. fluorescens* strain. It was concluded that MVOCs, and specifically DMDS, might be involved in the suppression of oncogenicity in plants (Dandurishvili et al., 2011). The main VOCs emitted by the *P. chlororaphis* strain 449 were 1-undecene, 2-nonanone, and 2-heptanone (**Figure 5**) along with and 2-undecanone (**Figure 2**) and lower amounts of DMDS. The composition of MVOCs produced by the *S. proteamaculans* 94 strain differed significantly from that emitted by *P. chlororaphis* strain 449, with DMDS being the main headspace MVOC emitted by the former (Popova et al., 2014). When these MVOCs were tested on the *A. tumefaciens* strain C58 and the cyanobacterium *Synechococcus* sp. strain PCC 7942, a strong *A. tumefaciens* bacteriostatic effect of DMDS (**25**) was confirmed and completely suppressed the growth of the cyanobacterium strain *Synechococcus* sp. 2-Nonanone and 2-heptanone (**Figure 5**) were effective on both microorganisms, whereas 2-undecanone completely inhibited the growth of *Synechococcus*, but did not appreciably affect *A. tumefaciens*. 1-Undecene (**Figure 5**) did not significantly affect the growth of any of the two microorganisms tested (Popova et al., 2014).

### MVOCs in Bacterial–Fungal Interactions

With respect to the functioning of soil microbial volatiles, most attention has been given to the suppressive effects of bacterial volatiles on soil eukaryotes that are harmful to agricultural crops; e.g., plant-pathogenic fungi (Zou et al., 2007; Verginer et al., 2010; Garbeva et al., 2014a,b). Rhizobacterial isolates comprising *S. plymuthica*, *S. odorifera*, *Stenotrophomonas maltophilia*, *Stenotrophomonas rhizophila*, *P. fluorescens*, and *P. trivialis* synthesize and emit complex blends of MVOCs that inhibit growth of many phytopathogenic and non-phytopathogenic fungi (Vespermann et al., 2007; Kai et al., 2010). The role of MVOCs in positive and antagonistic interactions between rhizobacteria and mycorrhizal fungi and their ecological significance has been described recently in an excellent review by Effmert et al. (2012).

**FIGURE 6:**
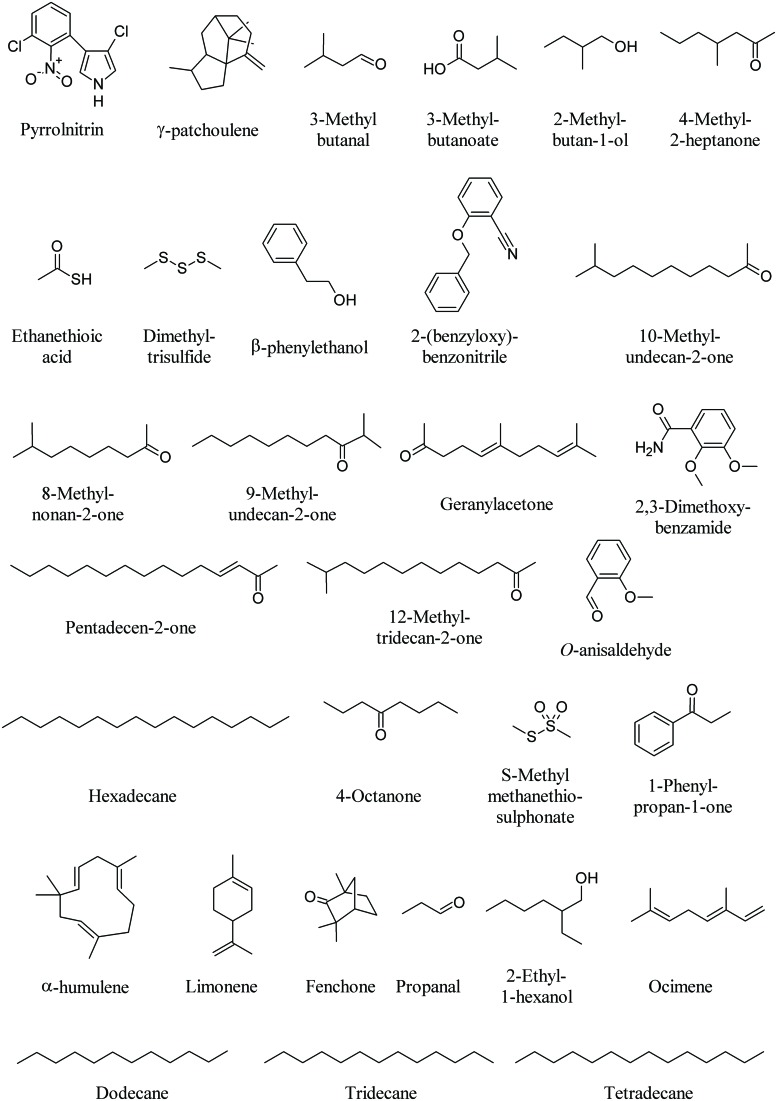
**Bacterial volatiles that interfere with fungi**.

One key antibiotic in soil is pyrrolnitrin (PRN, **Figure 6**). PRN is a chlorinated phenylpyrrol antibiotic that was first isolated from *Burkholderia pyrrocinia* and was later found in other genera, such as *Pseudomonas*, *Enterobacter*, *Myxococcus*, and *Serratia* (Garbeva et al., 2004). PRN has shown broad-spectrum activity against a range of fungi belonging to the Basidiomycota, Deuteromycota, and Ascomycota, including several economically important phytopathogens such as *Rhizoctonia solani*, *Botrytis cinerea*, *Verticillium dahliae*, and *Sclerotinia sclerotiorum*. For example, PRN production by *Burkholderia cepacia* strain 5.5B was related to the suppression of stem rot of poinsettia (*Euphorbia pulcherrima*) caused by *R. solani* (Hwang et al., 2002). PRN has been used as a lead structure in the development of a new phenylpyrrol agricultural fungicide (Ligon et al., 2000).

The mycelium of *Tuber borchii*, a commercial truﬄe species, is used as a model system for *in vitro* ectomycorrhizal synthesis, infected seedling production and biotechnological applications (see below for truﬄe volatiles). Bacteria with unusual biological activities could be a major problem during large-scale production of inoculum for truﬄe-infected seedling. For instance, a *Staphylococcus pasteuri* strain shows notable antifungal activity against *T. borchii* mycelium due to the production of MVOCs. Interesting molecules emitted by the fungal–bacterial interaction were γ-patchoulene, known for its antifungal activity, 3-methyl butanal (**Figure 6**) and 1-octen 3-ol (**Figure 1**). Typical metabolites of the *Staphylococcus* sp. were 2-undecanone (**Figure 2**), 2-nonanone (**Figure 5**), 3-methylbutanoate, 2-methylbutan-1-ol, 4-methyl-2-heptanone, ethanethioic acid, and dimethyl trisulfide (**Figure 6**; Barbieri et al., 2005).

A strong negative influence on the mycelial growth of the soil-borne phytopathogenic fungus *R. solani* (99–80%) was observed under the test conditions by MVOCs emitted by *Stenotrophomonas maltophilia* R3089, *Serratia plymuthica* HRO-C48, *Stenotrophomonas rhizophila* P69, *Serratia odorifera* 4Rx13, *Pseudomonas trivialis* 3Re2-7, *S. plymuthica* 3Re4-18, and *Bacillus subtilis* B2g. Although many of the bacterial VOCs could not be identified due to no matches being found with mass-spectra of volatiles in the databases, most of them were species-specific, and overlapping MVOCs patterns were found for *Serratia* sp. and *Pseudomonas* sp. (Kai et al., 2007). For example, β-phenylethanol could be detected in *S. rhizophila*, *S. epidermidis*, *S. plymuthica*, and *S. odorifera*, while other unidentified compounds were only emitted from pseudomonads, such as 2-(benzyloxy)benzonitrile (**Figure 6**; Kai et al., 2007).

The MVOC emission profile of *Xanthomonas campestris* pv. *vesicatoria* 85-10 consists of more than 50 compounds, the majority consisting of ketones and methylketones while the dominant compound is 10-methylundecan-2-one followed by 8-methylnonan-2-one, 9-methylundecan-2-one, geranylacetone, pentadecen-2-one, and 12-methyltridecan-2-one (**Figure 6**). However, when some of these compounds were tested on the fungus *R. solani* contradictory results were found with either promoting and inhibiting effects (Weise et al., 2012).

The volatile compounds produced by *Bacillus atrophaeus* CAB-1 include a range of alcohols, phenols, amines, and alkane amides. Hexadecane, 2,3-dimethoxybenzamide and *O*-anisaldehyde (**Figure 6**), were among the most abundant MVOCs. *O*-anisaldehyde was found to exert the highest inhibition on the mycelial growth of the fungal pathogen *Botrytis cinerea* (Zhang et al., 2013).

Significant growth inhibition of two phytopathogenic fungi, *R. solani* and *Alternaria alternata*, was observed with high concentrations of DMDS (**Figure 3**), 2-undecanone (**Figure 2**), dimethyl trisulfide, 4-octanone, *S*-methyl methanethiosulphonate, and 1-phenylpropan-1-one (**Figure 6**) emitted by *Burkholderia ambifaria* (Groenhagen et al., 2013). Fifteen *Burkholderia tropica* strains significantly inhibited the mycelial growth of four plant pathogenic fungi, *Colletotrichm gloesporioides, Fusarium culmorum, F. oxysporum*, and *Sclerotum rolffsi*. The volatile profile of *B. tropica* strain MTo431 showed the presence of several MVOCs know to play an important role in the antagonistic antifungal mechanism, including α-pinene (**Figure 1**), DMDS (**Figure 3**), ocimene, limonene, and fenchone (**Figure 6**; Tenorio-Salgado et al., 2013). *Bulkholderia gladioli* pv. *agaricola* strains produced MVOCs which inhibited fungal growth and reduced the growth rate of *F. oxysporum* and *R. solani*. Limonene was the most effective compound (Elshafie et al., 2012).

Several ectosymbiotic bacterial species live in association with fungi. The fungus *F. oxysporum* MSA 35 [wild-type (WT) strain] is a nonpathogenic *Fusarium* strain, which exhibits antagonistic activity to plant pathogenic *F. oxysporum* isolates (Minerdi et al., 2009). The fungus lives in association with a consortium of ectosymbiotic bacteria including *Serratia* sp. strain DM1 and *Achromobacter* sp. strain MM1. The WT strain, when cured of the bacterial symbionts, is pathogenic to lactuce, causing wilt symptoms similar to those of pathogenic *F. oxysporum* f. sp. *lactucae* (Minerdi et al., 2011). The major MVOC of *Achromobacter* sp. strain MM1 was DMDS, whereas *Serratia* sp. strain DM1 MVOC profile was made of DMDS, propanal, 2-ethyl-1-hexanol, dodecane, tridecane, and tetradecane (**Figure 6**). These volatiles had no effect on lettuce seedling growth. However, the WT strain of *F. oxysporum* MSA 35 produced a higher amount of α-humulene (**Figure 6**) and β-caryophyllene (**Figure 1**) with respect to cured *F. oxysporum*. β-Caryophyllene was found to be responsible for the lactuce growth promotion (Minerdi et al., 2011).

## Overview of Bioactive Fungal Volatiles

Several fungi show the ability to synthesize and emit MVOCs. Grass endophytic *Epichloë* species (Clavicipitaceae, Ascomycota; Schiestl et al., 2006), rust fungi including *Puccinia monoica* and *Uromyces pisi* (Pucciniaceae, Uredinales, Basidiomycota; Kaiser, 2006), truﬄes (*Tuber* sp., Pezizales, Ascomycota; Splivallo et al., 2011), some soil saprophytes (*Trichoderma* sp.; Yuan et al., 2012), mushrooms (sporocarps; Fraatz and Zom, 2010), fungi isolated from humid building materials (Schleibinger et al., 2008; Araki et al., 2009), wood and diseased plants (*Ceratocystis fimbriata*, Ascomycota) have the potential to generate MVOCs. Most of fungal MVOCs also exert either potent inhibitory or stimulatory effects on plants (Hung et al., 2013).

### MVOC of Fungal Endophytes

Endophytes constitute an important group of plant-associated fungal symbionts, which occur in both below-ground and above-ground tissues (Yuan et al., 2010; Waqas et al., 2014; Zhou et al., 2014). Volatile-producing endophytes (VPEs) may be of primary interest because of their production of antibiotic or pleasant MVOCs (Khan et al., 2014). From a biotechnological viewpoint, VPEs produce a broad spectrum of odorous compounds with different physicochemical and biological properties that make them useful in both industry and agriculture (Yuan et al., 2012).

**FIGURE 7:**
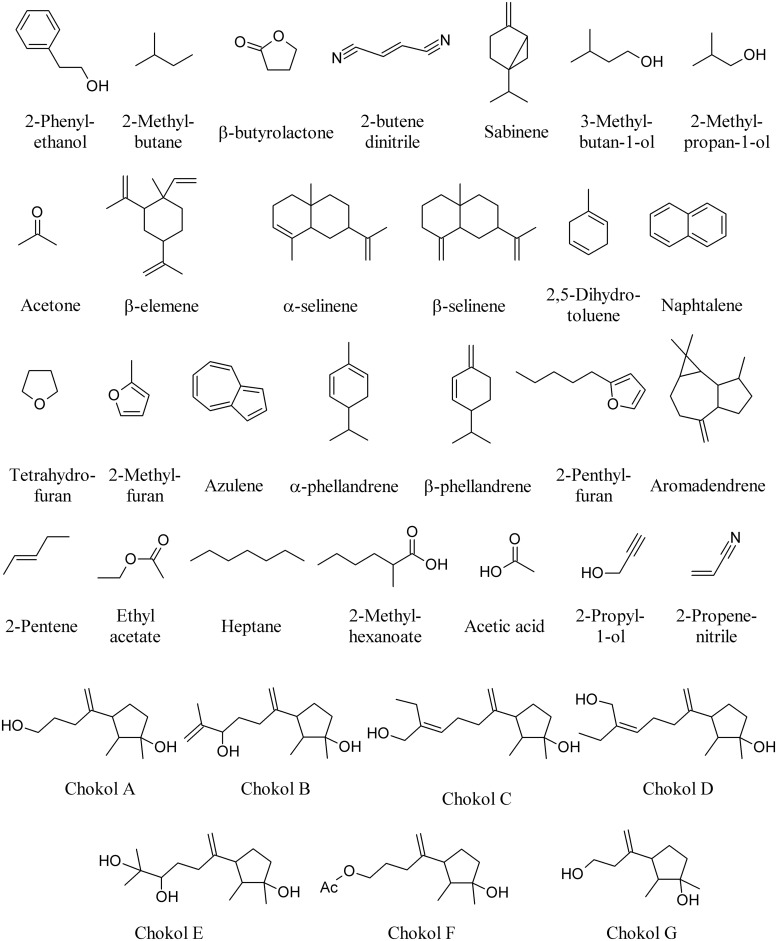
**Volatiles of fungal endophytes**.

Endophytic fungi of several Ascomycota lineages are capable of producing MVOCs, but members of the Xylariaceae family may be an especially rich source. *Aspergillus niger* produces 2-phenylethanol on the host plant *Rosa damascena* (Wani et al., 2010); *Botrytis* sp. BTF21 produces 2-methyl butane, β-butyrolactone, and 2-butene dinitrile on the host plant *Musa* sp. (Ting et al., 2010); *Phomopsis* sp. produces sabinene, 3-methylbutan-1-ol, 2-methylpropan-1-ol and acetone on the host plant *Odontoglossum* sp. (Singh et al., 2011); whereas *Nodulisporium* sp. produces β-elemene, α-selinene, β-selinene, and 2,5-dihydrotoluene (**Figure 7**) on the host plant *Cinnamomum loureirii* (Park et al., 2010).

Several species of *Muscodor* growing on different plant species produce different MVOCs including among others: naphthalene, tetrahydofuran, 2-methylfuran, azulene, α-phellandrene, β-phellandrene, 2-pentylfuran, aromadendrene (**Figure 7**), and β-caryophyllene (**Figure 1**) on different host plants such as *Actinidia chinensis*, *Ananas ananassoides*, *Ginkgo biloba*, and *Myristica fragrans* (Macias-Rubalcava et al., 2010; Yuan et al., 2012). The use of *Muscodor* MVOCs has been suggested as a promising option to replace methyl bromide fumigation as a means of controlling soil-borne plant diseases (Strobel, 2006). β-caryophyllene (**Figure 1**) is also emitted by the endophyte *Phialocephala fortinii* (Kramer and Abraham, 2012).

Among the least studied taxa of plant-associated fungal endophytes are the unspecialized, widespread soil-borne fungal endophytes belonging to the genus *Acremonium*. It was recently found that endophyte inoculated tomato plants emitted diverse terpenes and sesquiterpenes at significantly lower amounts as compared to endophyte free-plants, demonstrating that *Acremonium strictum* is able to induce changes in volatile emissions of the host plants (Jallow et al., 2008).

The fungal endophyte NRRL 50072, isolated from *Eucryphia cordifolia* in northern Patagonia, produces a variety of medium-chain and highly branched, with a third of the short- and medium-chain compounds also produced when cultures grow on a cellulose substrate. Among these are 2-pentene, 3-methylbutan-1-ol, ethyl acetate, heptane, and 2-methylhexanoate (**Figure 7**); collectively, these and other MVOCs have been highlighted for their potential as fuel alternatives as myco-diesel (Griffin et al., 2010).

Sesquiterpenes, chokols A–G (**Figure 7**) have been isolated from *Epichloe typhina* an endophytic fungus of *Phleum pratense*, and have been found to be fungitoxic to the leaf spot disease pathogen *Cladosporium phlei* (Kumar and Kaushik, 2012). Other enophytic fungi isolated from plum (*Prunus domestica*) leaves show antagonistic activity against *Monilinia fructicola*. Here, the most frequently isolated species is *Phaeosphaeria nodorum* and four isolates produced inhibitory volatiles to *M. fructicola*. The volatiles produced by these fungi were identified as ethyl acetate, 3-methylbutan-1-ol, acetic acid, 2-propyl-1-ol, and 2-propenenitrile (**Figure 7**). The fungal volatiles inhibited growth and reduced the width of the hyphae, and caused the disintegration of the hyphal content (Pimenta et al., 2012).

Current perspectives on the volatile-producing fungal endophytes have been recently reviewed (Yuan et al., 2012).

### MVOCs of Truﬄes

**FIGURE 8:**
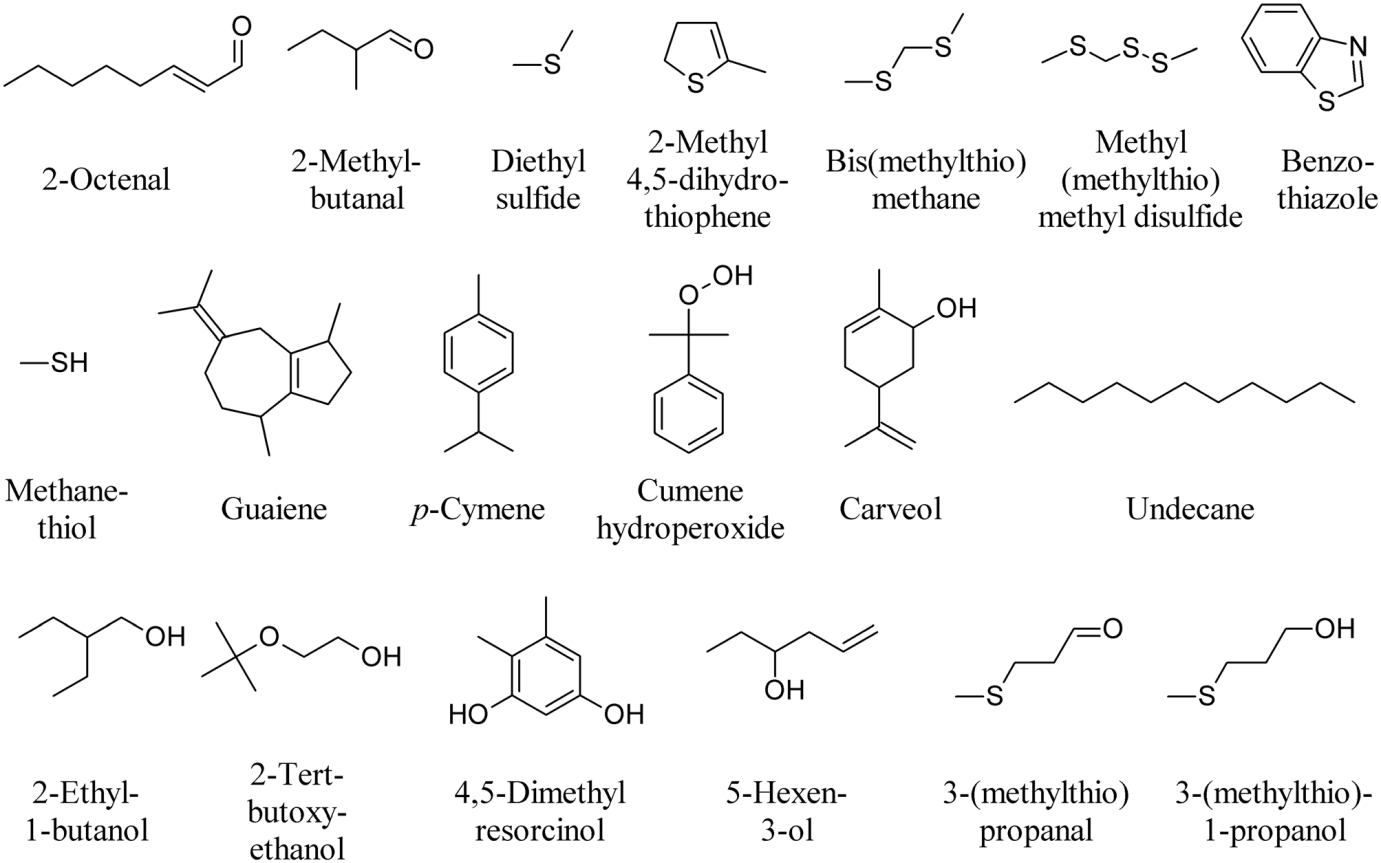
**Major volatiles emitted by truffles**.

Truﬄes use volatile signals throughout their life cycle to regulate their interactions with other organisms. Despite this fascinating aspect, the functional role of truﬄe volatiles in nature has rarely been investigated. In truﬄes, more than 200 VOCs have been described from various truﬄe species in the presymbiotic mycelial stage, during the mycorrhizal stage when the fungus enters a symbiosis with plant roots, and during the reproductive stage (Splivallo et al., 2011). The volatile profiles have been studied in the three most representative species: *Tuber melanosporum*, *T. magnatum*, and *T. borchii*. 2-Octenal (**Figure 8**) seems to be specific to symbiotic fungi as it has been reported in *T. borchii*, *T. melanosporum*, and *T. indicum* as well as in other mycorrhizal fungi (Splivallo et al., 2007). DMDS, 3-Methylbutanal (**Figure 6**), 2-methylbutanal, and dimethyl sulfide have also been found in most truﬄes investigated to date, while 2-methyl 4,5-dihydrothiophene has only been described from fruiting bodies of *T. borchii* (**Figure 8**; Splivallo et al., 2011). Bis(methylthio)methane, is the major contributor to the aroma of the white truﬄe *T. magnatum*, while other volatiles include dimethyl sulfide, methyl(methylthio)methyl disulfide, benzothiazole, methanethiol, and some terpenoids including, carveol, guaiene, *p*-cymene, cumene hydroperoxide (**Figure 8**) and limonene (**Figure 6**; Gioacchini et al., 2005, 2008). 1-Octen-3-ol (**Figure 1**) is produced by both truﬄe mycelium and fruiting bodies (Menotta et al., 2004; Splivallo et al., 2007). Mycelial cultures of *T. borchii* were found to emit camphor (**Figure 1**), *n*-undecane, 2-ethyl-1-butanol, 2-tert-butoxyethanol, 4,5-dimethyl resorcinol, 5-hexen-3-ol, and 3-(methylthio)propanal (**Figure 8**). Submerged fermentation colures of *T. melanosporum* were found to emit dimethyl sulfide, dimethyl trisulfide (**Figure 6**), DMDS, methanethiol, 3-(methylthio)propanal, and 3-(methylthio)-1-propanol (**Figure 8**; Liu et al., 2013).

### Bioactive MVOCs Emitted by Other Fungi

**FIGURE 9:**
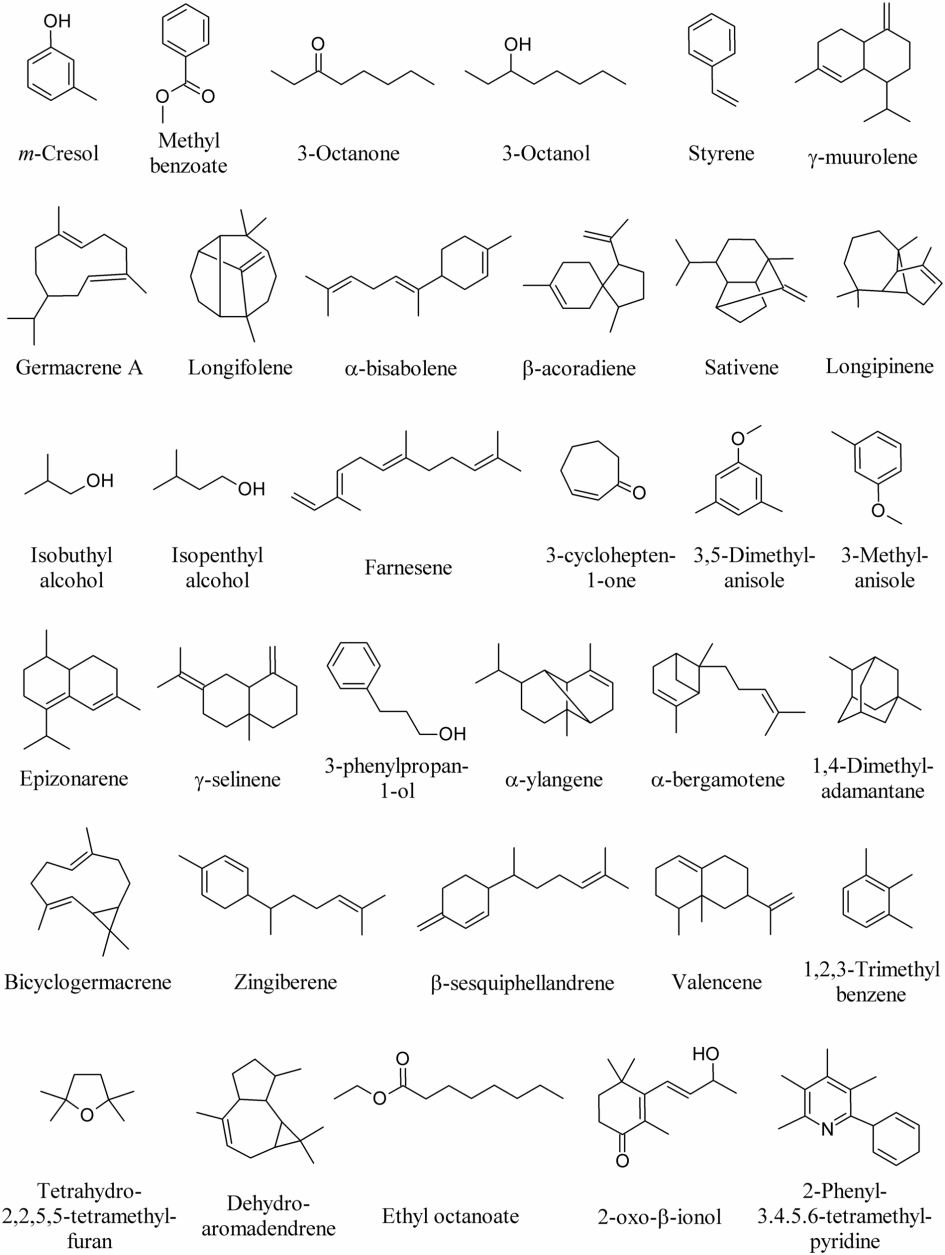
**Bioactive MVOCs produced by other fungi**.

Volatile organic compounds produced by fungi provide an alternative diagnostic approach for the identification of fungal strains (Zhang et al., 2014). Many other MVOCs have been identified from several fungal species, most of which exerting a significant effect on plants and plant-associated organisms. *m*-Cresol and methyl benzoate (**Figure 9**) were identified as major active volatile compounds from *Ampelomyces* sp. and *Cladosporium* sp., respectively, and found to elicit *Arabidopsis* ISR against the pathogen *Pseudomonas syringae* pv. *tomato* DC3000 (Naznin et al., 2014). 1-Octen-3-ol (**Figure 1**), 3-octanone, and 3-octanol (**Figure 9**) are emitted by the fungus *Fomes fomentarius* and found to induce contrasting behavior of the fungivorous beetle *Bolitophagus reticulatus* in olfactometer bioassays (Holighaus et al., 2014).

The pine weevil *Hylobius abietis* is a severe pest of conifer seedlings. The isolation of a fungus (*Penicillium expansum*) from feces and frass of *H. abietis* and the biological activity of its volatile metabolites styrene and 3-methylanisole showed that styrene significantly reduced male and female pine weevils’ attraction to cut pieces of Scots pine twigs, whereas 3-methyl anisole (**Figure 9**) only reduced male weevil attraction to pine twigs (Azeem et al., 2013).

Microbial volatile organic compounds emitted by *F. culmorum* and *Cochliobolus sativus* significantly decreased barley (*Hordeum vulgare*) leaf surface and mean root length. Among bioactive compounds emitted [including germacrene A, longifolene, α-bisabolene, and β-acoradiene], sativene was found the most emitted compound (**Figure 9**; Fiers et al., 2013).

Microbial volatile organic compounds of *Trichoderma viride* cultured on Petri plates in a shared atmosphere with *Arabidopsis* without direct physical contact, prompted plants with taller, bigger, and earlier flowered plants with more lateral roots. The most abundant MVOCs were isobutyl alcohol (**163**), isopentyl alcohol (**164**), and farnesene (**Figure 9**), along with the presence of 3-methylbutanal and geranylacetone (**Figure 6**; Hung et al., 2013).

Microbial volatile organic compounds were collected from the headspace of four ectomycorrhizal, three pathogenic, and two saprophytic fungi. Principal component and cluster analyses revealed that fungal species differ in their odor profiles, particularly in the pattern of sesquiterpenes. Among ectomycorrhizal fungi, 3,5-dimethyl anisole was uniquely released by *Laccaria bicolor*, whereas β-caryophyllene (**Figure 1**) and 3-cyclohepten-1-one were unique to *Paxillus involutus*. Among pathogenic fungi, longipinene was the unique sesquiterpene released by *Armillaria mellea*, which along with the other pathogens (*Pholiota squarrosa* and *Verticillium longisporum*) emitted geosmin (**Figure 1**), γ-muurolene, α-bisabolene, and epizonarene (**Figure 9**).

With regards to the terpenoid emission, the saprophytic species *Stropharia rugosoannulata* had unique emissions of α-muurolene (**Figure 4**), γ-selinene, and 3-phenylpropan-1-ol (**Figure 9**). *Trichoderma viride* was the only one releasing α-ylangene, α-bergamotene, bicyclogermacrene, zingiberene, β-sesquiphellandrene, and valencene (**Figure 9**; Müller et al., 2013). β-Caryophyllene (**Figure 1**) has been also detected in MVOCs emissions of saprophyte *F. oxysporum* (Campos et al., 2010), non-toxigenic strain of the saprophyte *Penicillium roqueforti* and the coprophyte *Coprinus cinereus* (Wihlborg et al., 2008), the pathogen *Phialophora fastigiata*, the mold *Penicillium caseifulvum*, and the saprophyte *Trichoderma pseudokoningii* (Müller et al., 2013). The production of many other fungal sesquiterpenes has been recently reviewed (Kramer and Abraham, 2012).

The potential roles of α-pinene, β-caryophyllene (**Figure 1**), tetrahydro-2,2,5,5-tetramethylfuran, dehydroaromadendrene, and sativene (**Figure 9**) produced by *Cladosporium cladosporioides* was evaluated on growth of tobacco seedlings *in vitro* when co-cultivated without physical contact (Paul and Park, 2013).

Microbial volatile organic compounds generated by *Streptomyces alboflavus* inhibit storage fungi *F. moniliforme*, *Aspergillus flavus*, *Aspergillus ochraceus*, *Aspergillus niger*, and *Penicillum citrinum* *in vitro*. The main MVOCs emitted by *S. alboflavus* fermentation broth were 2-methyl isoborneol (**Figure 1**), 1,4-dimethyladamantane and 1,2,3-trimethyl benzene (**Figure 9**; Wang et al., 2013).

The MVOCs produced by the yeast *Saccharomyces cerevisiae* strain CR-1 are able to inhibit the vegetative development of the fungus *Guignardia citricarpa*, causal agent of the disease citrus black spot. 3-Methylbutan-1-ol, 2-methylbutan-1-ol (**Figure 6**), 2-phenylethanol, ethyl acetate (**Figure 7**), and ethyl octanoate (**Figure 9**), which were naturally found in the atmosphere produced by the yeast, were found to considerably inhibit the mycelial development and interfered negatively with the production of the morphogenesis-related enzymes (Fialho et al., 2011).

Tea bushes entangled by rhizomorphs of *Marasmius crinisequi* are mostly devoid of leaves. This is due to the emission of a defoliation-inducing MVOC by the rhizomorphs. The MVOCs emitted by *M. crinisequi* were identified as 3-oxo-β-ionol and 2-phenyl-3,4,5,6-tetramethylpyridine (**Figure 9**; Su et al., 2011).

Microbial volatile organic compounds emitted by the yeast *Saccharomyces cerevisiae* were studied as a chemical control effectiveness of citrus black spot, caused by the fungus *G. citricarpa* at postharvest. Ethyl acetate, 2-methylbutan-1-ol (**Figure 6**), 3-methylbutan-1-ol, 2-phenylethanol (**Figure 7**), and ethyl octanoate (**Figure 9**) were the main MVOCs emitted. An artificial MVOCs mixture prepared on the basis of the composition of the above volatiles mimicked the inhibitory effects on *G. citricarpa* of the natural MVOCs released by *S. cerevisiae* (Fialho et al., 2010).

## MVOCs Role in Inducing Phenotypic Plant Responses

Considerable progress is also being made in understanding the important role ofMVOCs. Bacterial and/or fungalMVOCsmodulate plant growth and defense, interspecies interaction between plant, bacteria, fungi, and nematodes, play a role as attractants of natural enemies, as bio-control agents and find suitable applications as pest/insect/herbivore management (Leroy et al., 2011; Davis et al., 2013; Weise et al., 2013; D’Alessandro et al., 2014). These progressive studies on MVOCs illustrate their critical roles in multitrophic interactions and their importance in both the ecosystem and sustainable agriculture systems.

**FIGURE 10:**
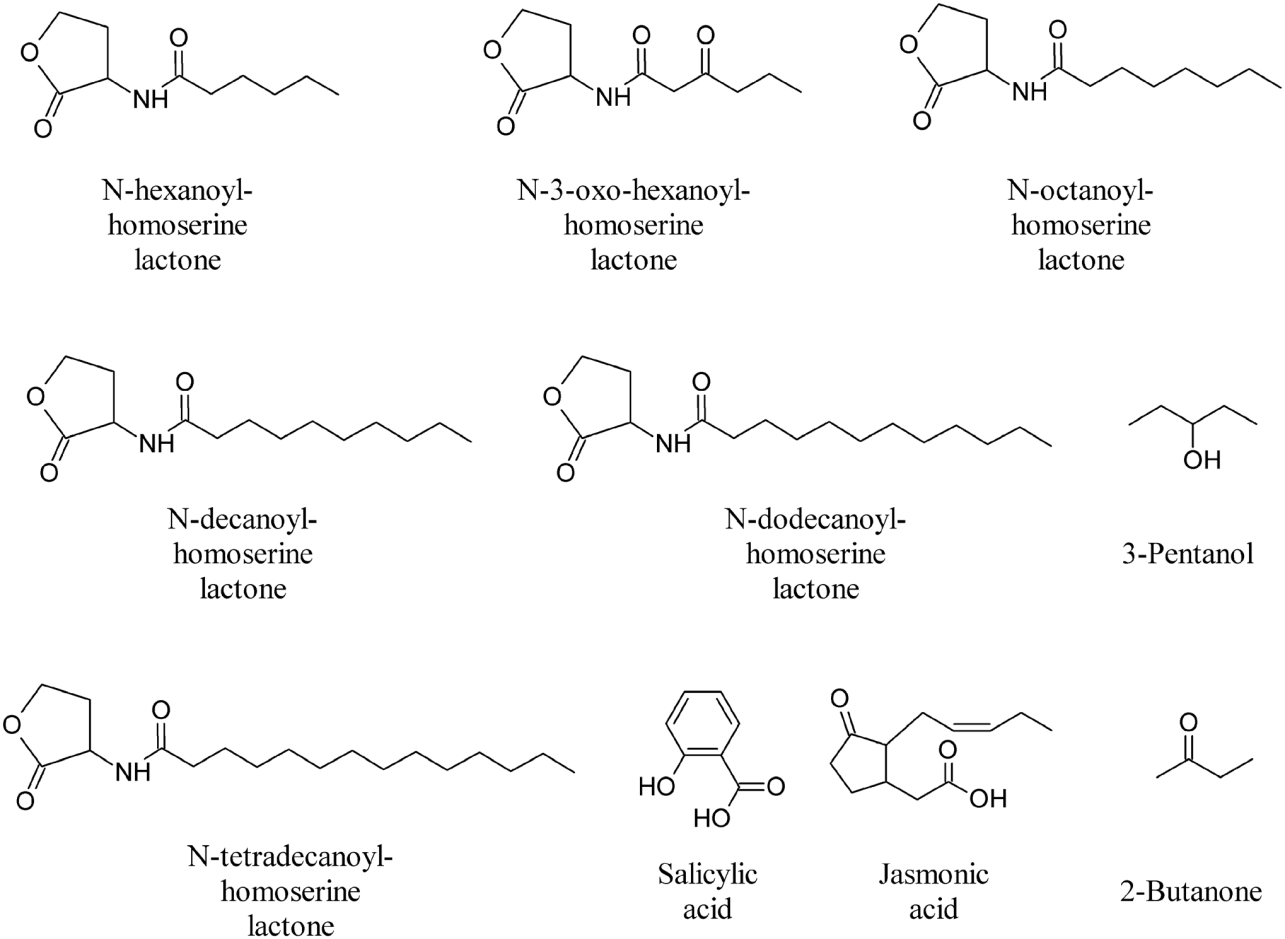
**Omoserine lactones and other bioactive volatiles**.

During the different stages of plant development extensive communication occurs between soil microorganisms and plants in which signal molecules from the two partners play important roles. MVOCs involved in multifaceted inter and intraspecific interactions, above and below ground, result in genetic, phenotypic and morphologic alteration of the interacting organisms (Effmert et al., 2012; Piechulla and Degenhardt, 2014; Penuelas et al., 2014). Fungal and bacterial species are able to detect the plant host and initiate their colonization strategies in the rhizosphere by producing canonical plant growth regulating substance such as auxins and/or cytokinins (Ortiz-Castro et al., 2009). Additional signals from microbes play a role in plant morphogenetic processes, as discussed in relation to AHLs. These compounds enable bacterial cells to regulate gene expression depending on population density (Ortiz-Castro et al., 2009). Very recently it was found that AHLs can be recognized by plants, alter gene expression in roots and shoots and modulate defense and cell growth responses (Ortiz-Castro et al., 2008; von Rad et al., 2008). In particular, medium (C_8_–C_14_)-chained AHL compounds (*N*-hexanoyl-homoserine lactone, *N*-3-oxo-hexanoyl-homoserine lactone, *N*-octanoyl-homoserine lactone, *N*-decanoyl-homoserine lactone, *N*-dodecanoyl-homoserine lactone, and *N*-tetradecanoyl-homoserine lactone, **Figure 10**) showed a dose-dependent effect on root architecture, altering primary root growth, lateral root formation, and root hair development of *Arabidopsis* (Ortiz-Castro et al., 2008; von Rad et al., 2008).

The majority of bacteria that activate ISR appear to do so via a salicylic acid (SA)-independent pathway involving jasmonate (JA) and ethylene signals (**Figure 10**). VOCs from *Bacillus amyloliquefaciens* strain IN937a triggered ISR through an ethylene-independent signaling pathway, whereas MVOCs from *Bacillus subtilis* strain GB03 appear, however, to operate through an ethylene-dependent pathway, albeit independent of the SA or JA signaling pathways (Ryu et al., 2004). This finding provides new insight into the role of MVOCs as initiators of defense responses in plants. Initially, in the process of developing an assay system to assess the growth promotion capacity of rhizobacteria *in vitro*, Ryu et al. (2003, 2004) found that bacterial volatiles are involved in plant growth promotion. An assessment of growth promotion induced by bacterial volatiles in *Arabidopsis* revealed that inoculation with the above mentioned GB03 or IN937a strains significantly promoted growth of *Arabidopsis*, as compared to water control or treatment with the *Escherichia coli* strain DH5α (Ryu et al., 2004). The two most abundant compounds released from cultures of strains GB03 and IN937a, albeit not from cultures of the other strains, were identified as 2,3-BD and its precursor acetoin (**Figure 3**; Ryu et al., 2003, 2004). The qualitative and quantitative compositions of volatile blends emitted by the growth-promoting strains differ significantly from those of the null growth-promoting *E. coli* strain DH5α (Ryu et al., 2003). Exogenous application of commercial acetoin and 2,3-BD result in the dose-dependent stimulation of plant growth, which simulates the effects of the volatile blend produced by the two *Bacillus* sp.

Microbial volatile organic compounds of plant growth promoting fungi (PGPF) Phoma sp. GS8-3 significantly enhanced the growth of tobacco seedlings (Naznin et al., 2014) and *Talaromyces wortmannii* FS2 MVOCs results in komatsuna (*Brassica campestris* L. var. *perviridis*) growth promotion and induced resistance against *Colletotrichum higginsianum* (Yamagiwa et al., 2011).

Priming the defense pathways with external signals enables the potentiated induction of defense response without immediately activating the defense signaling cascades that would be accompanied by energy expenditure for defense mobilization (Pare et al., 2005). In the case of PGPR priming of plant defenses, induction of the primed state is thought to result in an increase in the amount or activity of cellular components that play important roles in defense signaling; while this process is not associated with direct changes in gene expression in leaves (Lee et al., 2012). The priming activity of 2,3-B, thus reducing plant susceptibility to disease, has been confirmed in several studies (Ryu et al., 2004; Cortes-Barco et al., 2010a,b; Park et al., 2013; D’Alessandro et al., 2014). For example, in controlled environment tests, application 2,3-BD to the soil reduced the diseased leaf area of *Agrostis stolonifera* by 20–40% for the fungal pathogens *Microdochium nivale*, *R. solani*, or *Sclerotinia homoeocarpa* compared to the water control (Cortes-Barco et al., 2010b).

In a separate study, the application of 2,3-BD failed to elicit ISR against *Pseudomonas syringae* pv. *tabaci* but did induce the ISR response against *P. carotovora* subsp. *carotovora*, suggesting that different defensive cascades are elicited in response to different pathogens. The precursor acetoin was on the other hand shown to trigger ISR against *P. syringae* in *Arabidopsis* (Rudrappa et al., 2010). Application of 3-pentanol and 2-butanone (**Figure 10**) on cucumber seedlings consistently triggered plant systemic defense responses against *P. syringae* pv. *lachrymans*. These compounds induce gene expression of plant green leaf volatile signaling pathway to attract natural enemies of pests, an indirect defense strategy that protects plants from herbivores (Scala et al., 2013). Also these compounds do not affect plant growth but increase fruit yields and resulted, unexpectedly, in a significant increase in the number of ladybird beetles, *Coccinella septempunctata*, a natural enemy of aphids (Song and Ryu, 2013).

Microbial volatile organic compoundsof *Serratia plymuthica* and *Stenotrophomonas maltophilia* significantly inhibited growth and induced H_2_O_2_ production in *Arabidopsis* in dual culture. Expression studies performed with different timing revealed altered transcript levels for 889 genes and 655 genes in response to *S. plymuthica* or *S. maltophilia* volatiles, respectively. Furthermore, specifically volatile-responsive genes were significantly overlapped with those affected by abiotic stress and genes responsive to both treatments were enriched for W-box motifs in their promoters and transcription factors (ERF2, ZAT10, MYB73, and WRKY18). Interestingly, the susceptibility of *wrky18* mutant lines to volatiles was significantly delayed, suggesting an indispensable role for WRKY18 in bacterial volatile responses (Wenke et al., 2012).

Volatiles released from different microbial species ranging from Gram-negative and Gram-positive bacteria to fungi exert an effect on leaf starch metabolism. Surprisingly, all microbial species tested emitted MVOCs that strongly promoted starch accumulation in leaves of both mono-and dicotyledonous plants. Starch content in leaves of plants treated for two days with MVOCs was comparable with or even higher than that of reserve organs such as potato tubers. Transcriptome and enzyme activity analyses of potato leaves exposed to volatiles emitted by *Alternaria alternata* revealed that starch over-accumulation was accompanied by up-regulation of sucrose synthase, invertase inhibitors, starch synthase (SS) class III and IV, starch branching enzyme and glucose-6-phosphate transporter. This phenomenon, which was designated as MVOC-ISAP (MVOC-induced starch accumulation process), was also accompanied by down-regulation of acid invertase, plastidial thioredoxins, starch breakdown enzymes, proteins involved in internal amino acid provision and less well defined mechanisms involving a bacterial-type stringent response (Ezquer et al., 2010). Time-course analyses of starch accumulation in *Arabidopsis* leaves exposed to fungal MVOCs emitted by *A. alternata* also revealed stimulation of starch biosynthesis during illumination. The increase of starch content in illuminated leaves of MVOCs-treated *hy1/cry1*, *hy1/cry2*, and *hy1/cry1/cry2* *Arabidopsis* mutants was many-fold lower than that ofWT leaves, indicating that MVOCISAP is subjected to photoreceptor-mediated control. This phenomenon was inhibited by cordycepin and accompanied by drastic changes in the *Arabidopsis* transcriptome. The use of different *Arabidopsis* knockout mutants revealed that the magnitude of the MVOCs-induced starch accumulation was low in mutants impaired in SS classes III and IV and plastidial NADP-thioredoxin reductase C (NTRC). The overall data thus showed that *Arabidopsis* MVOC-ISAP involves a photo-controlled, transcriptionally and post-translationally regulated network wherein photoreceptor-, SSIII-, SSIV-, and NTRC-mediated changes in redox status of plastidial enzymes play important roles (Li et al., 2011). The discovery that microbial volatiles trigger starch accumulation enhancement in leaves constitutes an unreported mechanism for the elucidation of plant carbohydrate metabolism by microbes (Ezquer et al., 2010; Li et al., 2011).

**Table 1** summarizes the effect of microbial volatiles on plants, bacteria, and fungi.

**Table 1:** Summary of effects of microbial volatiles on plants, bacteria, and fungi.

Phylum	Species	Effective on	Effect	Molecule(s)	References
Bacteria	*Serratia marcescens * MG-1	Fungi and plants	Growth inhibition	N.d.	Vespermann et al. (2007)
Bacteria	*Stenotrophomanas maltophilia * R3089	Fungi and plants	Growth inhibition	N.d.	Vespermann et al. (2007)
Bacteria	*Stenotrophomanas rhizospehila * P69	Fungi and plants	Growth inhibition	N.d.	Vespermann et al. (2007)
Bacteria	*Pseudomanas aeruginosa * PAO1, PAO14, Tb, TBCF10839, and PUPa3	Plants	Growth inhibition	HCN	Blom et al. (2011)
Bacteria	*Pseudomanas trivialis * 3Re2-7	Plants	Growth inhibition	N.d.	Vespermann et al. (2007)
Bacteria	*Serratia odorifera*	Plants	Growth inhibition	Sodorifen	Vespermann et al. (2007)
Bacteria	*Serratia plymuthica * 3Re4-18, HRO-C48, IC14	Plants	Growth inhibition	N.d.	Vespermann et al. (2007), Blom et al. (2011)
Bacteria	*Pseudomanas fluorescens * A112	Plants	Growth inhibition (shoot and root)	N.d.	Åström and Gerhardson (1989)
Bacteria	Rhizosphere strains (more than 42 strains predominantly from *Burkholderia* genus)	Plants	Growth inhibition or promotion (dose dependent)	N.d.	Blom et al. (2011)
Bacteria	Rhizosphere strains (isolated from rhizosphere of lemon plants) L263, L266, L272a, L254, L265a, and L265b	Plants	Growth promoting and modulation of root architecture	Volatile mixture	Gutierrez-Luna et al. (2010)
Bacteria	*Arthobacter agilis* UMCV2	Plants	Growth promotion	*N,N*-dimethyl-hexadecanamine	Velazquez-Becerra et al. (2011)
Bacteria	*Bacillus megaterium* XTBG34	Plants	Growth promotion	2-Penthylfuran	Zou et al. (2010)
Bacteria	*Bacillus amyloliquefaciens * IN937a	Plants	Growth promotion and induced systemic resistance (ISR)	2,3-Butanediol; acetoin	Ryu et al. (2003, 2004)
Bacteria	*Bacillus subtilis* GBO3	Plants	Growth promotion and ISR	2,3-Butanediol; acetoin	Ryu et al. (2003, 2004)
Bacteria	*Pseudomanas chlororaphis* O6	Plants	Growth promotion, ISR, and drought stress tolerant	2,3-Butanediol	Han et al. (2006), Cho et al. (2008)
Fungi	*Muscodor yucatanensis*	Fungi and plants	Allelochemical effects against other endophytic fungi, phytopathogenic fungi, and plants	Mixture of volatile organic compounds (VOCs)	Macias-Rubalcava et al. (2010)
Fungi	*Muscodor albus*	Fungi and bacteria	Collectively they acted synergistically to kill a broad range of plant- and human-pathogenic fungi and bacteria	Isoamyl acetate	Strobel et al. (2011)
Fungi	*Muscodor crispans*	Fungi and bacteria	Effective against a wide range of plant pathogens, including the fungi *Pythium ultimum*, *Phytophthora cinnamomi*, *Sclerotinia sclerotiorum*, and *Mycosphaerella fijiensis* (the black sigatoka pathogen of bananas), and the serious bacterial pathogen of citrus, *Xanthomonas axonopodis* pv. *citri*. In addition, the VOCs of *M. crispans* killed several human pathogens, including *Yersinia pestis*, *Mycobacterium tuberculosis*, and *Staphylococcus aureus*.	Mixture of volatile compounds	Mitchell et al. (2010)
Fungi	*Tuber melanosporum, Tuber indicum*, and *Tuber borchii* (truﬄes)	Plants	Growth inhibition	2-Octenal	Splivallo et al. (2007)
Fungi	*Trichoderma virens*	Fungi and plants	Growth promotion and induction of defense responses of *Arabidopsis thaliana* against *Botrytis cinerea*	β-Caryophyllene; β-elemene; germacrene D; δ-cadinene	Angel Contreras-Cornejo et al. (2014)
Fungi	Mold fungi	Plants	Induced defense and protection against *Botrytis cinerea*	1-Octen-3-ol	Kishimoto et al. (2007)
Fungi	*Fusarium oxysporum * MSA 35	Plants	Induced shoot length, root length, and fresh weight of lettuce seedlings	β-Caryophyllene	Minerdi et al. (2011)
Fungi	*Phomopsis* sp.	Fungi	Possess antifungal properties and an artificial mixture of the VOCs mimicked the antibiotic effects of this organism with the greatest bioactivity against a wide range of plant pathogenic test fungi including: *Pythium, Phytophthora*, *Sclerotinia, Rhizoctonia*, *Fusarium, Botrytis*, *Verticillium*, and *Colletotrichum*.	Sabinene; isoamyla alcohol; 2-methyl propanol; 2-propanone	Singh et al. (2011)
Fungi	*Trametes gibbosa*	Fungi	Serves as attractant for fungus eating beetles	1-Octen-3-ol	Thakeow et al. (2008)
Fungi	*Trametes versicolor*	Fungi	Serves as attractant for fungus eating beetles	γ-Patchoulene; δ-cadinene; isoledene; β-guaiene	Drilling and Dettner (2009)
Fungi	*Phoma* sp.	Fungi	The volatiles of *Phoma* sp. possess antifungal and fuel properties	Unique mixture of VOCs, including a series of sesquiterpenoids, some alcohols, and several reduced naphthalene derivatives.	Strobel et al. (2011)
Fungi	*Muscodor albus*	Fungi and bacteria	Volatile mixture were effectively used to control postharvest plant diseases	2-Methyl butanol; isobutyric acid	Mercier and Jimenez (2004)

N.d., not determined.

## Exploiting MVOCs for Sustainable Crop Protection and Production

Diverse and rapidly evolving pathogens and global climate changes threaten the world crop yield and food security. The increased use of synthetic pesticides and fertilizers provides immediate solutions for the plant disease and crop yield problems, respectively, but in the end, they drastically affect human and environment health. Although bio-pesticides, bio-fertilizers, and bio-control agents derived from living microbes are becoming suitable replacements for the hazardous synthetic pesticides and fertilizers, their reduced efficiency, still high costs and inconstent field performance generally relegate them to niche products (Glare et al., 2012).

Over the past decade, research on MVOCs–plant interactions has led to an increasingly conceptual understanding of the intriguingly complex and dynamic nature of MVOCs, by stressing their potential role in enhancing crop protection and productivity in a sustainable way. As discussed above, exposing plants to MVOCs results in a significant modulation of plant metabolomics, physiology, and transcriptional status, which leads to the assumption that plants have the ability to perceive and respond toMVOCs. Most of the studies have, however, been conducted under lab conditions. Only recently have a few studies been performed in open field conditions to demonstrate efficient adoption of MVOCs for a sustainable crop protection and production (Cortes-Barco et al., 2010a,b; Song and Ryu, 2013). These studies clearly demonstrate the need for implementation of MVOCs application in open field conditions and stress their multiple roles to increase pathogen resistance, protection against herbivores and in general as bio-control agents. We now have the means to begin a new era of MVOCs applications for a sustainable crop protection and production strategies as a possible substitute for synthetic and hazardous chemical pesticides and fertilizers. Effective deployment of MVOCs still, however, remains a major challenge.

## Challenges for the Deployment of MVOCs under field conditions

Microbial volatile organic compounds as plant defense and growth modulators is still in its infancy. Up to now, only 10,000 microbial species described of the millions of species on Earth and only a 1000 MVOCs released by 400 bacteria and fungi have been described in the literature (Lemfack et al., 2014).

The effect of volatile compounds varies from lab to field conditions, as discussed in relation to 2,3-BD (**Figure 3**; Han et al., 2006; Cortes-Barco et al., 2010a,b; Song and Ryu, 2013). The contrasting results reported in the literature suggest that some of the MVOCs may modulate growth/defense in a species-dependent manner. For instance, 2,3-BD used at field conditions has been show to exert its effect only as a modulator of defense and none of the studies demonstrated its effect as growth modulator. Thus, before generalizing MVOCs as growth or defense modulators, it is necessary to evaluate either single MVOCs or MVOCs mixtures on different crop species both at lab and field conditions. Microbes produce a plethora of MVOCs and their effect on plant physiology is immense. This implies not only the need for application of single molecules, but also the experimentation of blends of differentMVOCs able tomodulate growth and defense of crop plants. Therefore, if a MVOCs mixture has an effect on plant resistance to diseases, it may at the same time exert a positive effect on plant growth and development. This means we will have an abundance of options available for fine-tuning blends of MVOCs.

Another challenging aspect is the manner of application of MVOCs and their exploitation in open field conditions. Most of MOVOCs have rapid evaporation rates, which makes them difficult to use at open field conditions. As discussed above, drench application of 2,3-BD, 3-pentanol, and 2-butanone (**Figure 10**) results in consistent reproducible field trails on a few crop plants (Cortes-Barco et al., 2010a,b; Song and Ryu, 2013), but we are still lacking an appropriate, durable method of MVOCs delivery in the field. Future studies are needed to understand better cost effective and efficient delivery of MVOCs.

Another concern on MVOCs use at field condition is the ‘allocation of fitness costs’ or ‘trade-off’; i.e., side effects of competing metabolic demands and requirement of energy and resources for the synthesis (Heil, 2001; Heil and Baldwin, 2002). MVOCs use for crop protection and productivity depend on the characterization of bioactive molecules, their proper bioactive dosage, and their role on plant growth and defense (Piechulla and Degenhardt, 2014). It should be considered that many MVOCs exert inhibitory effects; some of them are also toxic. However, after due assessing of the dose-response effect on specific crops their use can be safely managed.

Microbial volatile organic compounds can be applied at low concentration, are fully biodegradable and have no hazardous effects of the kind found in synthetic pesticides or fertilizers. In our opinion, the exploitation of MVOCs in the field as biocontrol agents in the field is only just evolving and its broad potential is only now beginning to be demonstrated (Song and Ryu, 2013). More open field studies and further physiological and molecular studies are required to show their full potential as substitutes and as potential candidates for a sustainable crop protection and production. When the most bioactive MVOCs are identified, cagedmolecules or pro-bioactive compounds that are readily degradable to bioactive MVOCs can be developed in chemical forms that allow handling, storing and safe delivering to crop fields.

## Concluding Remarks

Microbial volatile organic compounds form a bioactive interface between plants and a myriad of microorganisms above and below ground where most of the interactions take place. MVOCs are intriguingly complex and dynamic and understanding their ecology and evolution is the key to bioprospecting suitable tools for crop protection and production for sustainable agriculture perspective. New understanding of the importance of MVOCs for crop plants both at the lab and open field conditions will make possible to adopt and implement sustainable crop protection and to develop production strategies.

Many of the current insights on MVOCs have been carried out under lab conditions and on a limited number of microorganisms and molecules, but they have shown profound effects on growth, development, and defense system of plants. Numerous MVOCs contribute to these dynamic processes, leading to countless interactions between plants, antagonists, and mutualistic symbionts. For a better understanding of the role of MVOCs at field level, more studies should be conducted to provide further scientific evidence that can be used to assess the cost effective, ecofriendly, and sustainable use of naturally produced MVOCs for crop welfare.

**FIGURE 11:**
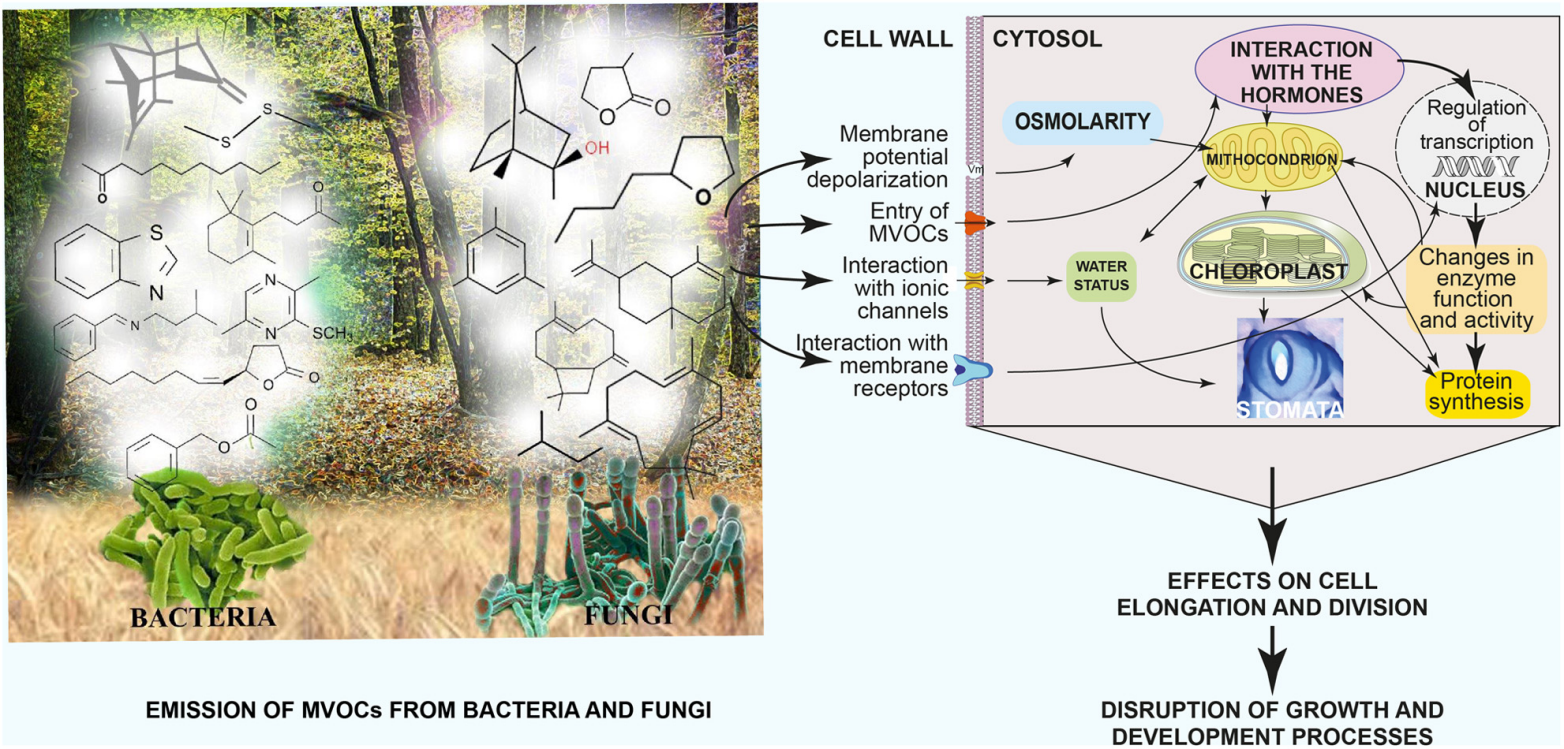
**Summary of differential emission of MVOCs by fungi and bacteria, their physiological effects on plant cells and the resulting effects on plant growth and productivity. Modified and adapted from Li et al. (2010)**.

**Figure 11** summarizes the differential emission of MVOCs by fungi and bacteria and the physiological effects of MVOCs on plant cells and their resulting outcomes on plant growth and productivity.

Among the next forthcoming research areas we foresee: expanding the knowledge on the MVOC biodiversity (by implementing the existing data bases), exploring the holistic action of MVOC mixtures with respect to single compound effects, establishing high-throughput analyses of plant responses to MVOCs, and producing non-volatile biodegradable precursors of bioactive MVOCs for an efficient delivery to crop fields.

## Conflict of Interest Statement

The authors declare that the research was conducted in the absence of any commercial or financial relationships that could be construed as a potential conflict of interest.
